# Isoniazid preventive therapy-related adverse events among Malawian adults on antiretroviral therapy: A cohort study

**DOI:** 10.1097/MD.0000000000030591

**Published:** 2022-09-30

**Authors:** Lufina Tsirizani-Galileya, Elasma Milanzi, Randy Mungwira, Titus Divala, Jane Mallewa, Donnie Mategula, Nginache Nampota, Victor Mwapasa, Andrea Buchwald, Matthew B. Laurens, Miriam K. Laufer, Joep J. Van Oosterhout

**Affiliations:** a Blantyre Malaria Project, Kamuzu University of Health Sciences, Blantyre, Malawi; b Melbourne School of Population and Global Health, University of Melbourne, Melbourne, Australia; c Department of Medicine, Kamuzu University of Health Sciences, Blantyre, Malawi; d Malawi-Liverpool-Wellcome Trust Clinical Research Programme, Blantyre, Malawi; e Liverpool School of Tropical Medicine, Liverpool, United Kingdom; f School of Public Health and Family Medicine, Kamuzu University of Health Sciences, Blantyre, Malawi; g Center for Vaccine Development and Global Health, University of Maryland School of Medicine, Baltimore, MD, USA; h Dignitas International, Zomba, Malawi; i Partners in Hope, Lilongwe, Malawi & Division of Infectious Diseases, David Geffen School of Medicine, University of California, Los Angeles, CA, USA.

**Keywords:** adverse events, antiretroviral therapy, human immunodeficiency virus, isoniazid preventive therapy, Malawi, tuberculosis

## Abstract

Adverse events may be a cause of observed poor completion of isoniazid preventive therapy (IPT) among people living with HIV in high tuberculosis burden areas. Data on IPT-related adverse events (AE) from sub-Saharan Africa are scarce. We report IPT-related AEs, associated clinical characteristics, and IPT discontinuations in adults who were stable on antiretroviral therapy (ART) when they initiated IPT. Cohort study nested within a randomized, controlled, clinical trial of cotrimoxazole and chloroquine prophylaxis in Malawians aged ≥ 18 years and virologically suppressed on ART. Eight hundred sixty-nine patients were followed for a median of 6 months after IPT initiation. IPT relatedness of AEs was determined retrospectively with the World Health Organization case-causality tool. Frailty survival regression modeling identified factors associated with time to first probably IPT-related AE. The overall IPT-related AE incidence rate was 1.1/person year of observation. IPT relatedness was mostly uncertain and few AEs were severe. Most common were liver and hematological toxicities. Higher age increased risk of a probably IPT-related AE (aHR = 1.02; 95% CI 1.00–1.06; *P* = .06) and higher weight reduced this risk (aHR = 0.98; 95% CI 0.96–1.00; *P* = .03). Of 869 patients, 114 (13%) discontinued IPT and 94/114 (82%) discontinuations occurred at the time of a possibly or probably IPT-related AE. We observed a high incidence of mostly mild IPT-related AEs among individuals who were stable on ART. More than 1 in 8 persons discontinued IPT. These findings inform strategies to improve implementation of IPT in adults on ART, including close monitoring of groups at higher risk of IPT-related AEs.

## 1. Introduction

Human immunodeficiency virus (HIV) infection increases the risk of developing tuberculosis (TB) 20-fold, and TB is the leading cause of death among persons living with HIV (PLHIV).^[1]^ In 2019, 81% of all HIV-associated TB deaths occurred in Africa^[2]^ and in Malawi around half of TB cases are associated with HIV.^[3,4]^

Because isoniazid preventative therapy (IPT) reduces TB disease risk by 35% in PLHIV,^[5]^ the World Health Organization (WHO) recommends IPT use in this population in Africa and other areas with high TB burden.^[1]^ Despite its proven efficacy, IPT coverage remains sub-optimal: 2.3 of the 6 million eligible PLHIV in high TB burden areas were on IPT in 2020.^[2]^ Where IPT has been implemented, drug adherence and completion of therapy are disappointingly low.^[6–8]^ Adverse events (AEs) of the drug can lead to premature discontinuation of IPT,^[9]^ contributing to decreased IPT effectiveness,^[10]^ but high-quality data on IPT toxicity from sub-Saharan Africa are sparse, in particular from populations on antiretroviral therapy (ART). Most previous studies focused on understanding the determinants of isoniazid induced hepatotoxicity,^[6,11,12]^ and limited information is available from African countries on other AEs, such as peripheral neuropathy, skin rash, vomiting, diarrhea, anemia, agranulocytosis, thrombocytopenia, and pellagra.^[10,13]^

To provide more insight into the frequency, variety and impact of AEs related to IPT in African PLHIV, we describe detailed AEs in a Malawian study population that initiated IPT while on well-established ART. We also examined possible risk factors for IPT-related AEs and evaluated the effect of AEs on IPT discontinuation.

## 2. Methods

### 2.1. Study design

We conducted a cohort study nested within a randomized, controlled, open-label trial comparing daily co-trimoxazole, weekly chloroquine, or no prophylaxis in adults aged 18 years and above. The main study was approved by the College of Medicine Research Ethics Committee and the University of Maryland Institutional Review Board. Study protocol and overall results of the clinical trial have been published previously.^[14,15]^ In summary, participants were recruited if they provided written informed consent and had been on ART for at least 6 months with CD4 > 250 cells/mm^3^ and viral load < 400 copies/mL.

Exclusion criteria included severe acute illness, history of hypersensitivity to study drugs, and abnormal blood parameters including serum creatinine > 3.3 mg/dL (men), or 2.7 mg/dL (women) and alanine aminotransferase (ALT) > 210 U/L (men) or 160 U/L (women). Routine 1 to 3-monthly follow up visits took place with intercurrent review in case of any illness symptoms. Data on AEs, including presenting symptoms, physical examination details, and laboratory monitoring results were routinely collected during clinical trial follow up and were graded using the National Institutes of Health, Division of AIDS toxicity tables.^[16]^

For the current cohort study, we included 869 patients who initiated IPT in 2017, when national HIV management guidelines introduced life-long IPT for patients on ART in the 5 highest TB burden districts of Malawi.^[17]^ All patients had been on long-term ART and were well established on the assigned study medication.

Participants initiated once daily oral isoniazid 300 mg and pyridoxine 25 mg if they had no TB symptoms and no known isoniazid allergy. Data on the following variables were extracted at the start of IPT or the last previous measured value (for laboratory measurements), ranging from the same day isoniazid treatment began to a maximum of 259 days previous: body weight, ART regimen, age, sex, CD4 count, viral load, ALT, and creatinine. Causality of adverse events was prospectively evaluated by clinicians during the parent trial, based on clinical impression. To determine AE relatedness to IPT formally, we retrospectively used the WHO standardized case-causality assessment tool.^[18]^ This tool defines the following AE relatedness categories: *Certainly related*: positive re-challenge; AE with plausible time relationship with drug intake; plausible response to drug withdrawal; cannot be explained by other drug/disease; event definitive pharmacologically and phenomenologically. *Probably related*: AE with plausible time relationship with drug intake; plausible response to drug withdrawal; unlikely attributed to other drug/disease. *Possibly related*: could also be explained by disease or other drug; information on drug withdrawal lacking or unclear. *Unlikely related*: disease/other drugs give plausible explanation; time relationship between AE and drug intake makes relationship improbable. *Conditional*: more information needed. *Unclassifiable*: inadequate information. Because we did not routinely re-challenge with isoniazid after a related AE, we could not assign the classification certainly IPT-related. For the purposes of this article, we were interested in identifying the risk of AEs that were probably related to isoniazid treatment. Two independent reviewers assessed and categorized AEs using the tool and discussed discrepant cases to reach agreement. Severity was graded using Division of AIDS toxicity tables,^[16]^ with mild, moderate, severe, life threatening, and leading to death.

### 2.2. Risk of experiencing an AE probably related to IPT

To investigate the association between time to a probably IPT-related AE and patient characteristics, we used frailty proportional hazards models, which allow for more than 1 sequential AE per participant. Follow-up time was calculated from initiation of isoniazid to either the time of an AE probably related to isoniazid or discontinuation of isoniazid or, if no AE occurred and isoniazid was not discontinued, the last recorded visit.

Individuals who experienced more than 1 probably IPT-related AE contributed multiple observations to the model with follow-up time for potential second observations beginning the day after first probably IPT-related AE. Patient characteristics examined for association with having a probably IPT-related AE included variables that were dichotomous (sex, viral load [<400 or ≥400 copies/mL], serum creatinine [<1.2 or > 1.2 mg/dL], serum ALT [<44 or > 44 units/mL], ART regimen [tenofovir/lamivudine/efavirenz or other]), categorical (study randomization arm [chloroquine, trimethoprim-sulfamethoxazole, or no therapy], age [<35, 36–49, or 50–85 years], body mass index [BMI; <18.5, 18.5–25, or 25–43], CD4 count [<250, 250–400, >400 cells/mm^3^], and continuous (weight [kg]). Patient characteristics associated with probably IPT-related AEs at α = 0.1 were included in a multivariate frailty proportional hazards model to obtain adjusted hazard ratios (aHR). AE failure rate was defined as the rate of occurrence of probably IPT-related AE, expressed as the average number of AEs per participant, accounting for censored observations.

### 2.3. Risk of discontinuation of IPT

We examined factors related to discontinuation of IPT using Cox proportional hazards models. For this analysis, participants contributed 1 observation each, with follow-up time beginning at first IPT initiation and ending either at discontinuation (failure) or end of study (censored). Patient characteristics examined for association with discontinuation of IPT were the same as for the association with a probably IPT-related AE.

We also examined whether having an AE was associated with risk of IPT discontinuation by including AE as a predictor in proportional hazards models. AE was included as a categorical variable and categorized as: participant had no AEs; participant had only AEs classified as unlikely related to IPT; or participant had at least 1 AE possibly or probably related to IPT. Patient characteristics associated with IPT discontinuation at α = 0.1 were included in a multivariate proportional hazards model to obtain aHRs.

## 3. Results

From March to November 2017, 869 participants initiated IPT and were included in the analysis. Fourteen individuals discontinued and re-started IPT. We accrued 548 person years of observation and the median follow-up time was 252 days (inter-quartile range: 195, 259). Most participants (664; 76%) were female. At IPT initiation, median age was 41 years (range: 21–85) and 54% were between 36 and 49 years of age; 9.6% had low BMI (<18.5 kg/m^2^), 58% had normal BMI (18.5–25 kg/m^2^), and 33% had BMI >25 kg/m^2^. The median CD4 count was 522 cells/µL (range: 167–1709) and in 3.6% it was < 250 cells/µL; 95% had viral load < 400 copies/mL. The large majority (97%) was on first-line ART with a fixed drug combination of tenofovir, lamivudine, and efavirenz. Serum creatinine and alanine transferase levels were within the normal range for 99% and 94% of participants, respectively. Details of baseline characteristics among all patients and patients who experienced AEs are shown in Table 1.

**Table 1:** Baseline characteristics of all participants and those who experienced possibly or probably isoniazid (IPT)-related adverse events (AEs), all expressed as N (%).

Variable	Baseline characteristics of all participants	Patient characteristics of individuals with AEs probably related to IPT*	Patient characteristics of individuals with AEs possibly related to IPT*
Total	869	80	259
Randomization arm			
Chloroquine	283 (32.6%)	20 (25%)	78 (30.1%)
TS	291 (33.5%)	30 (37.5%)	93 (35.9%)
No therapy	295 (34%)	30 (37.5%)	88 (34%)
Age (yr)			
≤35	227 (26.1%)	18 (22.5%)	65 (25.1%)
36–49	466 (53.6%)	39 (48.8%)	140 (54.1%)
50–85	175 (20.1%)	22 (27.5%)	54 (20.9%)
Missing	1 (0.1%)	1 (1.3%)	0
Sex			
Female	664 (76.4%)	66 (82.5%)	191 (73.8%)
Male	205 (23.6%)	14 (17.5%)	68 (26.3%)
BMI (kg/cm^2^)			
<18.5	83 (9.6%)	11 (13.8%)	26 (10%)
18.5–25	501 (57.7%)	50 (62.5%)	155 (59.9%)
25–43	285 (32.8%)	19 (23.8%)	78 (30.1%)
CD4 count (cells/microliter)			
<250	31 (3.6%)	5 (6.3%)	9 (3.5%)
250–400	190 (21.9%)	22 (27.5%)	58 (22.4%)
>400	648 (74.6%)	53 (66.3%)	192 (74.1%)
Viral load (copies/mL)			
<400	829 (95.4%)	78 (97.5%)	247 (95.4%)
≥400	40 (4.6%)	2 (2.5%)	12 (4.6%)
Serum creatinine (mg/dL)			
Normal (<1.2)	860 (99.0%)	79 (98.8%)	256 (98.8%)
Raised (≥1.2)	9 (1.0%)	1 (1.3%)	3 (1.2%)
Serum ALT (units/L)			
Normal (<44)	820 (94.4%)	76 (95%)	246 (95%)
Raised (≥44)	49 (5.6%)	4 (5%)	13 (5%)
ART regimen			
TDF/3TC/EFV	839 (96.6%)	76 (95%)	253 (97.7%)
Other	30 (3.5%)	4 (5%)	6 (2.3%)

3TC = lamivudine, ALT = alanine aminotransferase, ART = antiretroviral therapy, BMI = body mass index, EFV = efavirenz, TDF = tenofovir disoproxil fumarate, TS = trimethoprim sulfamethoxazole.

* Seventeen individuals had both possibly related and probably related AEs and are included among probably related.

### 3.1. IPT-related adverse events

Most participants (685/869; 79%) experienced at least 1 AE after initiating IPT, with a total of 1726 recorded AEs between March 2017 and November 2018. Using the WHO case-causality tool, we determined that 422 of the 1726 (24%) recorded AEs were possibly or probably IPT-related (Table 2). Among all participants, 340/869 (39%) experienced at least 1 possibly or probably IPT-related AE.

**Table 2:** Adverse events: relatedness to IPT, severity, and outcomes.

AE category	Total	Grade 1 and 2, mild or moderate	Grade 3 and 4, severe or life threatening	Grade 5, death	Ongoing at end of follow up	Resolved
Probably related*	102 (5.9%)	95 (93.1%)	7 (6.9%)	0 (0%)	13 (12.8%)	89 (87.3%)
Possibly related*	320 (18.5%)	308 (96.3%)	11 (3.4%)	1 (0.3%)	188 (58.8%)	131 (40.9%)
Unlikely related*	1304 (75.6%)	1269 (97.3%)	35 (2.7%)	0 (0%)	265 (20.3%)	1039 (79.7%)
Total	**1726 (100%**)	**1672 (96.9%**)	**53 (3.1%**)	**1 (0.1%**)	**466 (27.0%**)	**1259 (72.9%**)

AE, adverse event; IPT, isoniazid preventive therapy.

* WHO causality tool^[16]^ classifications (classifications 1, 5, and 6 were not encountered): 1. Certainly related: positive re-challenge; AE with plausible time relationship with drug intake; plausible response to drug withdrawal; cannot be explained by other drug/disease; event definitive pharmacologically and phenomenologically. 2. Probably related: AE with plausible time relationship with drug intake; plausible response to drug withdrawal; unlikely attributed to other drug/disease. 3. Possibly related: could also be explained by disease or other drug; information on drug withdrawal lacking or unclear. 4. Unlikely related: disease/other drugs give plausible explanation; time relationship between AE and drug intake makes relationship improbable. 5. Conditional: more information needed. 6. Unclassifiable: inadequate information.

The 1726 AEs included 18 possibly or probably IPT-related AEs with grade 3 and 4 severity, 10 of which were serious (life-threatening [4] or leading to either incapacitation [1], hospitalization [4], or death [1]). The most common IPT-related AEs of grade 1 and 2 severity were peripheral neuropathy, headache, diarrhea, pellagra, vomiting, confusion, neutropenia, gynecomastia, and elevated ALT. A total of 43 serious AEs occurred during follow up, 3 of which were probably related to IPT. Details of the type of IPT-related AEs are included in Table 3. One death was classified as possibly related to IPT in a participant who developed paresthesia followed by quadriplegia and possible respiratory insufficiency.

**Table 3:** Common adverse events possibly or probably related to IPT.

Adverse event	Possibly related to IPT N (%)	Probably related to IPT N (%)
**Total**	**320**	**102**
Raised ALT	120 (37.6)	7 (6.8)
Neutropenia	31 (9.7)	4 (3.9)
Anemia	30 (9.4)	2 (1.9)
Peripheral neuropathy	19 (6.0)	11 (10.7)
Gastroenteritis	17 (5.3)	10 (9.7)
Musculoskeletal pain	10 (3.1)	3 (2.9)
Leukopenia	12 (3.8)	0 (0)
Dizziness	3 (0.9)	8 (7.8)
Malaise	6 (1.9)	3 (2.9)
Rash	3 (0.9)	6 (5.8)

ALT = alanine aminotransferase, IPT = isoniazid preventive therapy.

### 3.2. Risk factors for adverse events probably related to IPT

For survival analysis, there were 959 observations among 869 individuals, with a median of 250 days of follow-up time per observation. AEs probably related to IPT occurred among 80 individuals: 57, 20, and 2 individuals had 1, 2, or 3 probably IPT-related AEs, respectively. No participant experienced 4 or more probably IPT-related AEs. The failure rate from Kaplan–Meier survival analysis, defined as the rate of experiencing a probably related AE in the cohort, including all follow-up time accounting for multiple observations and censoring, was 12% (95% CI: 9%−14%).

In frailty survival models, age and weight were associated with time to having a probably IPT-related AE. For individuals with the same frailty, increasing age by 1 year was associated with a 2% increase in probably IPT-related AE hazard: aHR 1.02; 95% CI 1.00–1.04; *P* = .06. A 1 kg increase in weight was associated with a 2% decrease (aHR 0.98; 95% CI 0.96–1.00; *P* = .03). Sex, ALT level, CD4 count, viral load, and ART regimen before the start of IPT were not significantly associated with experiencing probably IPT-related AEs (Table 4).

**Table 4:** AE failure rate and crude and adjusted hazard ratios of experiencing an adverse event probably related to IPT by patient characteristic from frailty proportional hazards models.

Patient characteristic	AE failure rate (95% CI)*	Crude hazard ratio (95% CI)	*P* value	Adjusted hazard ratio (95% CI)	*P* value
Total population	0.12 (0.09, 0.14)				
Randomization arm			.27		
Chloroquine	0.09 (0.06, 0.13)	0.68 (0.39, 1.18)			
TS	0.12 (0.09, 0.16)	0.95 (0.57, 1.58)			
No therapy	0.13 (0.09, 0.17)	1 (REF)			
Age (yr)		1.02 (1.00, 1.04)	.06	1.02 (1.00, 1.04)	.06
Sex			.16		
Male	0.08 (0.04, 0.12)	1 (REF)			
Female	0.13 (0.1, 0.15)	1.46 (0.83, 2.57)			
Weight (kg)		0.98 (0.96, 1.00)	.03	0.98 (0.96, 1.00)	.03
BMI (kg/cm^2^)			.07		
<18.5	0.18 (0.1, 0.26)	2.14 (1.02, 4.48)			
18.5–25	0.13 (0.1, 0.16)	1.55 (0.92, 2.61)			
25–43	0.08 (0.05, 0.11)	1 (REF)			
CD4 count (cells/microliter)			.31		
<250	0.15 (0.03, 0.28)	1.73 (0.61, 4.85)			
250–400	0.14 (0.09, 0.2)	1.28 (0.77, 2.12)			
>400	0.11 (0.08, 0.13)	1 (REF)			
Viral load (copies/mL)			.22		
<400	0.12 (0.1, 0.14)	2.37 (0.55, 10.26)			
≥400	0.05 (0.00, 0.12)	1 (REF)			
Serum ALT (units/L)			.60		
Normal (<44))	0.12 (0.09, 0.14)	1.24 (0.46, 3.37)			
Raised ALT (≥44)	0.1 (0.02, 0.18)	1 (REF)			
ART			.52		
TDF/3TC/EFV	0.12 (0.09, 0.14)	0.74 (0.24, 2.25)			
Other	0.13 (0.01, 0.26)	1 (REF)			

3TC = lamivudine, AE = adverse event, ALT = alanine aminotransferase, ART = antiretroviral therapy, BMI = body mass index, CI = confidence interval, EFV = efavirenz, REF = reference, TDF = tenofovir disoproxil fumarate, TS = trimethoprim sulfamethoxazole.

* AE failure rate was defined as the rate of occurrence of probably IPT-related AE, expressed as the average number of AEs per participant, accounting for censored observations.

### 3.3. Risk of discontinuation of IPT

Of all participants, 112 (13%) discontinued IPT and 85/112 (76%) had at least 1 AE possibly or probably related to IPT, 11 (13%) being mild, 69 (81%) moderate, and 5 (6%) severe. Of the remaining 27/114 (24%) individuals who discontinued IPT, 9 had AEs unrelated to IPT and 18 had no AE. Having at least 1 AE possibly or probably related to IPT was associated with an increased risk of IPT discontinuation (aHR 2.44; 95% CI 1.5–4.1; *P* < .0001) compared to individuals with no AEs (Table 5). Being on tenofovir/lamivudine/efavirenz was associated with a reduced risk of IPT discontinuation compared to being on a different ART regimen (aHR 0.35; 95% CI 0.18, 0.71; *P* = .003). Few patients who had discontinued IPT (14/112; 13%) restarted after reassurance by clinicians.

**Table 5:** Crude and adjusted hazard ratios of discontinuing IPT by patient characteristic from proportional hazards models.

Patient characteristic	Crude hazard ratio (95% CI)	*P* value	Adjusted hazard ratio (95% CI)	*P* value
AE during follow-up		<.0001		<.0001
None	1 (REF)		1 (REF)	
AEs unrelated to IPT	0.24 (0.11, 0.53)		0.23 (0.11, 0.52)	
AEs possibly or probably related to IPT	2.61 (1.57, 4.33)		2.44 (1.46, 4.06)	
Randomization arm		.05		.12
Chloroquine	0.57 (0.35, 0.92)		0.6 (0.36, 0.98)	
TS	0.98 (0.64, 1.49)		0.86 (0.56, 1.31)	
No therapy	1 (REF)		1 (REF)	
Age (yr)	1.02 (1, 1.04)	.09		
Sex		.73		
Male	1 (REF)			
Female	1.08 (0.69, 1.69)			
Weight (kg)	0.98 (0.96, 1)	.01		
BMI (kg/cm^2^)		.01		.18
<18.5	2.18 (1.12, 4.24)		1.55 (0.78, 3.08)	
18.5–25	1.94 (1.21, 3.09)		1.56 (0.97, 2.52)	
25–43	1 (REF)		1 (REF)	
CD4 count (cells/microliter)		.01		.11
<250	2.58 (1.24, 5.37)		1.94 (0.91, 4.1)	
250–400	1.52 (1, 2.31)		1.4 (0.91, 2.13)	
>400	1 (REF)		1 (REF)	
Viral load		.59		
Non-detectable	1.31 (0.48, 3.56)			
Detectable	1 (REF)			
Serum ALT (units/L)		.86		
Normal (<44)	1.07 (0.47, 2.45)			
Raised ALT (≥44)	1 (REF)			
ART		.003		.003
TDF/3TC/EFV	0.36 (0.18, 0.71)		0.35 (0.18, 0.71)	
Other	1 (REF)		1 (REF)	

3TC = lamivudine, AE = adverse event, ALT = alanine aminotransferase, ART = antiretroviral therapy, BMI = body mass index, CI = confidence interval, EFV = efavirenz, REF = reference, TDF = tenofovir disoproxil fumarate, TS = trimethoprim sulfamethoxazole.

## 4. Discussion

We followed a large and well characterized cohort of adult PLHIV on established and successful ART who initiated IPT with a median follow up of 6 months. Close to 40% experienced at least 1 IPT-related AE. The vast majority (97%) of all AEs were of mild or moderate severity and only 6% were considered probably associated with IPT.

Characteristics that increased risk of experiencing an AE that was probably related to IPT were older age and lower body weight. Nearly 1 in 8 persons discontinued IPT, mostly in the presence of an AE, despite limited severity and uncertain relatedness with IPT in most cases.

The risk factors for IPT-related AEs that we identified are consistent with those of similar studies in both resource limited and high resource settings.^[2,19,20]^ As is the case with medication toxicity in general, higher age increased risk of developing IPT-related AEs.

Decreases in metabolic enzyme activity and total body water occur with increasing age. These factors reduce drug clearance and the volume of distribution of hydrophilic drugs such as isoniazid, leading to increased drug concentrations.^[13,21,22]^ The association of lower weight with higher risk of developing IPT-related AEs could be due to the fact that people with lower lean body mass (LBM) have a decreased volume of distribution for isoniazid.^[23–25]^ In adults ≥ 35 kg, guidelines do not recommend IPT dose adjustment for body weight or LBM and persons with lower LBM will exhibit higher drug concentrations, increasing their risk of toxicity.^[17]^ We did not identify an association between baseline liver enzyme abnormalities and risk of IPT-related AEs, reported in other studies.^[21,26]^ However, we only assessed ALT (not other liver enzymes) and may therefore have underestimated hepatotoxicity. The number of participants with elevated ALT was very low, possibly related to our study population being stable on longer-term ART and hepatotoxicity findings may be different in patients who initiate ART and IPT at the same time.

Our study showed an IPT discontinuation rate of 13%. This is consistent with some but not all studies in similar settings. In an Eritrean study of persons on nevirapine- and efavirenz-based ART, 3.8% discontinued IPT,^[12]^ and in a Ugandan study 11%.^[27]^ Both studies followed patients who initiated IPT when already established on ART. One study in urban^[8]^ and 1 in rural^[28]^ Malawi found higher discontinuations (both 25%) than in our study. In the former study, around 60% of enrolled participants were established on ART, mainly stavudine/lamivudine/nevirapine, while in the latter, 16% were on ART at IPT initiation and 69% initiated ART (regimens not reported) at some point during IPT. AEs that occur in the dynamic period after ART initiation are common and may be attributed to IPT if initiated concurrently, when distinction from toxicities by antiretroviral and other drugs, and from Immune Reconstitution Inflammatory Syndrome and incident opportunistic infections can be difficult. Risk factors for discontinuation of IPT were presence of a possibly or probably IPT-related AE and being on an ART regimen other than the standard first line treatment. Regimens containing zidovudine and protease inhibitors have AEs such as peripheral neuropathy and liver enzyme elevation. Since these AEs can be difficult to distinguish from IPT-related effects, clinicians may attribute them to isoniazid and may then decide to discontinue IPT.

Strengths of our study are the large and well-characterized cohort with consistent and detailed follow up. Patients were on stable, long-term ART, making incorrect attribution of AEs to antiretroviral medications less likely, but reducing generalizability of the findings to populations initiating ART and IPT concurrently. A limitation of our study was that we did not routinely re-challenge participants who discontinued IPT, which is a requirement to determine if relatedness of AEs with IPT is certain, so we could not assign that classification. Secondly, the study population was from a single, urban site and had pre-specified inclusion and exclusion criteria from the main clinical trial, reducing generalizability of our results. The WHO causality tool was implemented retrospectively, therefore our conclusions about relatedness may differ from those of the clinicians who cared for the patients and made ad hoc decisions on continuation or discontinuation of IPT. Lastly, because this was a secondary analysis and N-acetyl transferase-2 genotyping was not performed in the main study, we did not have this information in order to distinguish between slow, intermediate, and fast acetylators. Acetylator status determines the rate of isoniazid elimination and increased isoniazid exposure in slow acetylators may have led to more IPT-related AEs.^[29]^

In conclusion, we found that among adults on established ART IPT-related AEs were common, but were mostly non-severe and often had uncertain relatedness to IPT. Older age and lower weight are risk factors for developing IPT-related AEs in Malawian adults on ART. Discontinuation of IPT occurred in 1 in 8 persons, generally in the presence of an IPT-related AE. Because few large-scale assessments of adverse reactions to IPT among individuals on ART exist in sub-Saharan Africa, these findings inform strategies to improve implementation of IPT in adults on ART. Intensified AE counseling at initiation of IPT may prioritize groups at higher risk of IPT-related AEs, in particular individuals with lower weight and higher age.

## Author contributions

**Conceptualization:** Lufina Tsirizani-Galileya, Randy Mungwira, Titus Divala, Matthew B. Laurens, Miriam K. Laufer, Joep J. van Oosterhout.

**Data curation:** Nginache Nampota, Miriam K. Laufer.

**Formal analysis:** Lufina Tsirizani-Galileya, Elasma Milanzi, Donnie Mategula, Andrea Buchwald.

**Methodology:** Nginache Nampota, Joep J. van Oosterhout.

**Supervision:** Joep J. van Oosterhout.

**Writing – original draft:** Lufina Tsirizani-Galileya.

**Writing – review & editing:** Lufina Tsirizani-Galileya, Elasma Milanzi, Randy Mungwira, Titus Divala, Jane Mallewa, Donnie Mategula, Nginache Nampota, Victor Mwapasa, Andrea Buchwald, Matthew B. Laurens, Miriam K. Laufer, Joep J. van Oosterhout.
